# Supplementary material for: HIVprotI: an integrated web based platform for prediction and design of HIV proteins inhibitors
Source: J Cheminform. 2018 Mar 9;10:12. doi: 10.1186/s13321-018-0266-y (PMC5845081; doi:10.1186/s13321-018-0266-y)
Supplement: Supplementary file 2 — Additional file 2. Source code of HIVProtI web server. [file 13321_2018_266_MOESM2_ESM.zip › HIVprotI_Source-code/hivprotI/weka-3-6-6/doc/weka/core/neighboursearch/CoverTree.CoverTreeNode.html]

CoverTree.CoverTreeNode


---


|  |  |  |
| --- | --- | --- |
| **Overview** \| **Package** \| **Class** \| **Tree** \| **Deprecated** \| **Index** \| **Help** \| **Weka's home** | |  |
| **PREV CLASS**   **NEXT CLASS** | **FRAMES**    **NO FRAMES**     **All Classes** |  |
| SUMMARY: NESTED \| FIELD \| CONSTR \| METHOD | DETAIL: FIELD \| CONSTR \| METHOD |  |


---


## weka.core.neighboursearch Class CoverTree.CoverTreeNode

```
java.lang.Object
  weka.core.neighboursearch.CoverTree.CoverTreeNode
```

**All Implemented Interfaces:**: java.io.Serializable, RevisionHandler

**Enclosing class:**: CoverTree

---

``` public class CoverTree.CoverTreeNode extends java.lang.Object implements java.io.Serializable, RevisionHandler ```

class representing a node of the cover tree.

**Version:**
:   $Revision: 1.4 $

**Author:**
:   Ashraf M. Kibriya (amk14[at-the-rate]cs[dot]waikato[dot]ac[dot]nz)

**See Also:**: Serialized Form

---

**Constructor Summary**

`CoverTree.CoverTreeNode()`
  
          Constructor for the class.

`CoverTree.CoverTreeNode(java.lang.Integer i,
double md,
double pd,
Stack<CoverTree.CoverTreeNode> childs,
int numchilds,
int s)`
  
          Constructor.


| **Method Summary** | |
| --- | --- |
| `java.lang.String` | `getRevision()`             Returns the revision string. |
| `boolean` | `isALeaf()`             Returns whether if the node is a leaf or not. |
| `Instance` | `p()`             Returns the instance represented by the node. |

**Methods inherited from class java.lang.Object**

`equals, getClass, hashCode, notify, notifyAll, toString, wait, wait, wait`

**Constructor Detail**

### CoverTree.CoverTreeNode

```
public CoverTree.CoverTreeNode()
```

:   Constructor for the class.

---


### CoverTree.CoverTreeNode

```
public CoverTree.CoverTreeNode(java.lang.Integer i,
                               double md,
                               double pd,
                               Stack<CoverTree.CoverTreeNode> childs,
                               int numchilds,
                               int s)
```

:   Constructor.

    **Parameters:**: `i` - The index of the Instance this node is associated with.: `md` - The distance of the furthest descendant.: `pd` - The distance of the node to its parent.: `childs` - Children of the node in a stack.: `numchilds` - The number of children of the node.: `s` - The scale/level of the node in the tree.


**Method Detail**

### p

```
public Instance p()
```

:   Returns the instance represented by the node.

    :   **Returns:**: The instance represented by the node.

---


### isALeaf

```
public boolean isALeaf()
```

:   Returns whether if the node is a leaf or not.

    :   **Returns:**: true if the node is a leaf node.

---


### getRevision

```
public java.lang.String getRevision()
```

:   Returns the revision string.

    :   **Specified by:**: `getRevision` in interface `RevisionHandler`
    :   **Returns:**: the revision


---


|  |  |  |
| --- | --- | --- |
| **Overview** \| **Package** \| **Class** \| **Tree** \| **Deprecated** \| **Index** \| **Help** \| **Weka's home** | |  |
| **PREV CLASS**   **NEXT CLASS** | **FRAMES**    **NO FRAMES**     **All Classes** |  |
| SUMMARY: NESTED \| FIELD \| CONSTR \| METHOD | DETAIL: FIELD \| CONSTR \| METHOD |  |


---
